# Antibacterial activity of high-dose nitric oxide against pulmonary *Mycobacterium abscessus* disease

**DOI:** 10.1099/acmi.0.000154

**Published:** 2020-08-10

**Authors:** Kristijan Bogdanovski, Trisha Chau, Chevalia J. Robinson, Sandra D. MacDonald, Ann M. Peterson, Christine M. Mashek, Windy A. Wallin, Mark Rimkus, Frederick Montgomery, Joas Lucas da Silva, Shashank Gupta, Abdi Ghaffari, Adrian M. Zelazny, Kenneth N. Olivier

**Affiliations:** ^1^​ Laboratory of Chronic Airway Infection, Pulmonary Branch, National Heart, Lung, and Blood Institute, National Institutes of Health, Bethesda, MD, USA; ^2^​ Nursing Department, Clinical Center, National Institutes of Health, Bethesda, MD, USA; ^3^​ Critical Care Therapy Section, Clinical Center, National Institutes of Health, Bethesda, USA; ^4^​ Beyond Air, Garden City, NY, USA; ^5^​ Department of Laboratory Medicine, Clinical Center, National Institutes of Health, Bethesda, MD, USA

**Keywords:** nontuberculous mycobacteria, *Mycobacterium abscessus*, nitric oxide, antibacterial activity, drug discovery

## Abstract

**Introduction:**

*
Mycobacterium abscessus* is an emerging pulmonary pathogen with limited treatment options. Nitric oxide (NO) demonstrates antibacterial activity against various bacterial species, including mycobacteria. In this study, we evaluated the effect of adjunctive inhaled NO therapy, using a novel NO generator, in a CF patient with pulmonary *M. abscessus* disease, and examined heterogeneity of response to NO *in vitro*.

**Methods:**

In the compassionate-use treatment, a 24-year-old CF patient with pulmonary *M. abscessus* was treated with two courses of adjunctive intermittent NO, first at 160 p.p.m. for 21 days and subsequently by escalating the dose up to 240 p.p.m. for 8 days. Methemoglobin, pulmonary function, 6 min walk distance (6MWD), qualify of life and sputum microbiology were assessed. *In vitro* susceptibility tests were performed against patient’s isolate and comparison clinical isolates and quantified by Hill’s slopes calculated from time–kill curves.

**Results:**

*
M. abscessus* lung infection eradication was not achieved, but improvements in selected qualify of life domains, lung function and 6MWD were observed during the study. Inhaled NO was well tolerated at 160 p.p.m. Dosing at 240 p.p.m. was stopped due to adverse symptoms, although methemoglobin levels remained within safety thresholds. *In vitro* susceptibility tests showed a dose-dependent NO effect on *M. abscessus* susceptibility and significant heterogeneity in response between *M. abscessus* clinical isolates. The patient’s isolate was found to be the least susceptible strain *in vitro*.

**Conclusion:**

These results demonstrate heterogeneity in *M. abscessus* susceptibility to NO and suggest that longer treatment regimens could be required to see the reduction or eradication of more resistant pulmonary strains.

## Introduction

*Mycobacterium abscessus* is a pathogenic nontuberculous mycobacteria (NTM) species and the most common cause of rapidly growing mycobacterial lung infection worldwide in patients with chronic inflammatory lung diseases such as cystic fibrosis (CF), non-CF bronchiectasis and chronic obstructive pulmonary disease (COPD) [1, 2]. Antibiotic therapy often fails to achieve complete eradication and no combination drug regimen reliably cures *M. abscessus* lung infection at this time [3, 4]. Therefore, there is an unmet need for the development of novel anti-NTM therapies.

Nitric oxide (NO) is a naturally produced lipophilic free radical that plays an essential role in host defence mechanisms against infection at various sites, including the lungs [5, 6]. It is generated by nitric oxide synthases (NOSs) utilizing l-arginine as the substrate, and molecular oxygen and reduced nicotinamide adenine dinucleotide phosphate (NADPH) as co-substrates [7]. Among host cells that are protective against pulmonary pathogens, macrophages play a pivotal role in the immune response against pulmonary mycobacterial infection. Studies suggest that NO production is a key determinant of macrophage activity against mycobacterial infection [8]. During infection, NO production is increased in alveolar macrophages as a result of bacterial products or inflammatory stimuli [9, 10]. Treatment with NOS inhibitors or knocking out NOS significantly enhanced the severity of infection in preclinical models [11–13]. Evidence shows that reduced airway NO levels in CF patients are associated with poor outcome [14, 15]. However, treatment of CF patients with low-dose NO (<40 p.p.m.) or attempts to boost endogenous NO production in lungs by l-arginine (NO precursor) supplementation had limited effect on outcome [16, 17]. Preclinical studies revealed that high-dose NO gas (≥160 p.p.m.) demonstrates broad-spectrum antibacterial activity [18, 19]. Miller *et al*. showed that intermittent high-dose inhaled NO (160 p.p.m.) treatment decreases *Pseudomonas aeruginosa* load in a rat model of lung infection [20]. Preliminary clinical studies revealed that intermittent inhaled NO therapy at higher doses (160 p.p.m.) is safe and well tolerated by healthy individuals as well as CF patients [21, 22]. In CF patients with pulmonary NTM disease, reductions in lung *M. abscessus* burden and improvement of airway function were observed after administration of intermittent NO at 160 p.p.m. [23, 24].

The objective of this study is to evaluate the effect of adjunctive inhaled NO therapy, by escalating dose up to 250 p.p.m., as compassionate treatment in a CF patient with chronic and progressive pulmonary *M. abscessus* disease. In addition, we examined the heterogeneity of response to high-dose NO in this patient’s isolate relative to other clinical *M. abscessus* isolates by performing *in vitro* susceptibility tests using a previously described NO exposure chamber [25].

## Methods

### Clinical treatment protocol

A 24-year-old CF patient with an 8-year history of *M. abscessus* subspecies *abscessus* refractory to treatment with multiple drug combinations associated with considerable toxicity was evaluated for treatment under an IRB- and FDA-approved expanded access treatment protocol for administration of intermittent, inhaled NO (https://clinicalstudies.info.nih.gov/ProtocolDetails.aspx?id=2017-H-0169). The patient had progressive deterioration in lung function, functional status and quality of life, and was denied lung transplantation consideration at multiple centres in the USA and Canada due to chronic *M. abscessus* lung infection.

The patient was admitted to the NIH Clinical Center for each treatment course and following signed informed consent had screening/baseline assessment, which included routine haematology, chemistry and liver function tests, venous methemoglobin (MetHb) level, pulmonary function and 6 min walk testing prior to NO administration. The patient was monitored for toxicity before and after inhalation with measurement of vital signs and continuously during each inhalation period with measurement of oxyhaemoglobin and MetHb saturation (Radical-7, Masimo Corp., CA, USA). Delivered and ambient concentrations of O_2_, NO and nitrogen dioxide (NO_2_) were also monitored using AeroNOx monitor (International Biomedical, Ltd, TX, USA). Efficacy measures included health-related quality of life [Cystic Fibrosis Questionnaire – Revised (CFQ-R) containing both generic and CF-specific functions [26]], lung function, 6MWD, inflammatory markers and sputum specimen processing in a microbiology laboratory to assess *M. abscessus* growth. The patient was assessed monthly for 4 months after the last NO treatment.

### NO delivery system and regimens

The NO delivery system was composed of a prototype NO generator device (Beyond Air, Garden City, NY, USA) and a patient delivery circuit (Fig. S1, available in the online version of this article). In brief, the generator uses plasma arc technology to produce NO from filtered room air. NO is directed through a NO_2_ filter (Sofnolime, Molecular Products, Louisville, CO, USA) and combined with room air so that the desired NO concentration is delivered to patients at 15 l min^−1^ and an FiO_2_ of 0.21. The patient circuit used a 2.4 m coaxial tubing with integral monitoring line delivering to a twin-ported CPAP mask secured with a four-point silicone harness (Intersurgical Ltd, Berkshire, UK). Based on previous preclinical and clinical studies [18–22], the planned treatment for course 1 was 160 p.p.m. inhaled NO for 30 min every 3–4 h daily (five times/day) for 1–14 days followed by three times per day for days 15–21 (Fig. S2). Planned treatment course 2 assessed maximum tolerated dose delivered in 30 min cycles to potentially enhance mycobacterial killing. Dose titration day 1 : 160 p.p.m. followed by increase in 10 p.p.m. for subsequent doses administered every 3–4 h through 200 p.p.m. Day 2 : 200 p.p.m. followed by increase in 10 p.p.m. for subsequent doses through 240 p.p.m. Days 3–21: maximum tolerated dose delivered in 30 min periods every 3–4 h, five times per day (Fig. S2).

### Bacterial strains

The clinical strains of *M. abscessus* were provided by the Microbiology Service, Department of Laboratory Medicine (DLM), National Institutes of Health (NIH) Clinical Center. A clinical isolate collected from this patient prior to NO inhalation along with previously published clinical strains of *M. abscessus* subspecies *massiliense* (MABm), collected from different time points in a CF patient’s clinical course, which showed progressive antibiotic resistance correlating with increasing antibiotic exposure were tested for *in vitro* susceptibility to NO (Table 1) [27–29]. Mycobacterial culture, concentration and preservation were described in detail previously [28].

**Table 1.** Clinical *M. abscessus* isolates tested for *in vitro* NO susceptibility

***M. abscessus*** Isolate #	Subspecies	Patient source	Clinical time point	Morphology
MAB-110917–1505	*abscessus*	Current report	Late infection prior to NO Rx	Rough
MAB-062600–1635	*massiliense*	Outbreak [25–27]	Early infection, clinical stability	Smooth
MAB-030804–1651	*massiliense*	Outbreak [25–27]	4 years after, clinical stability	Smooth
MAB-010708–1655	*massiliense*	Outbreak [25–27]	8 years after initial isolate, post-rapid clinical decline	Rough

### Time–kill assays

The mycobacterial culture was prepared, as previously described, by diluting frozen stocks in artificial sputum at a density of 10^6^ colony-forming units (c.f.u.) ml^−1^ and placing 2 ml in each well of a six-well plate [30]. To assess the sensitivity of the patient’s *M. abscessus* strain and comparison isolates to exogenous NO, we performed *in vitro* susceptibility assays using the previously described NO exposure chamber (Fig. S3) [25]. We used an artificial sputum media designed to mimic CF patient sputum and to reduce intra- and inter-patient variability of real sputum used in preclinical models [30]. The basic artificial sputum medium (pH 7.0) consists of 0.5 g mucin from pig stomach mucosa (NBS Biologicals, Huntingdon, UK), 0.4 g DNA (Fluka), 0.59 mg diethylene triamine pentaacetic acid (Sigma), 0.5 g NaCl, 0.22 g KCl, 0.5 ml egg yolk emulsion (Oxoid) and 0.5 g casamino acids (Difco) 100 ml^−1^ water. The uptake of exogenous NO gas into the artificial sputum liquid phase was assessed by the accumulation of NO_2_/NO_3_ (byproducts of NO). The nitric oxide uptake in artificial sputum was measured by analysing NO_2_/NO_3_ concentrations with the Nitrate/Nitrite Colorimetric Assay kit (Cayman, Ann Arbor, MI, USA) as per the manufacturer’s guidelines. Linear regression analysis of NO_2_/NO_3_ accumulation versus time demonstrated a consistent and predictable delivery of NO to the bacterial culture media (Fig. S4). Mycobacterial culture plates were placed inside the NO chamber and treated with humidified air (control) or increasing concentrations of NO (160, 250, 300, 400 p.p.m.) for up to 10 h. At 2 h time intervals, aliquots were taken from both control and NO-treated bacteria cultures to quantify the size of bacterial population. Serial 10-fold dilutions were performed in 0.85% sterile saline and plated on Middlebrook 7H11 agar (Becton Dickinson, Franklin Lakes, NJ, USA). Plates were incubated for 5 days at 37 °C for colony counts in c.f.u. ml^−1^. At least three independent time–kill assays were performed for each bacterial strain and NO concentration.

### Time–kill curve fitting and statistical analysis

The bacterial viability data derived from time–kill assays were analysed in GraphPad Prism 7.0 (GraphPad, Inc., San Diego, CA, USA). Log c.f.u. counts were plotted against time for each experiment. A sigmoidal dose–response model (variable slope) was fitted to the time–kill data to determine the Hill’s slope for each strain [31]. The Mann–Whitney test was used to calculate the significance of differences between the Hill’s slopes of the *M. abscessus* strains.

## Result

### Clinical results

#### Treatment course 1

**Fig. 1.**
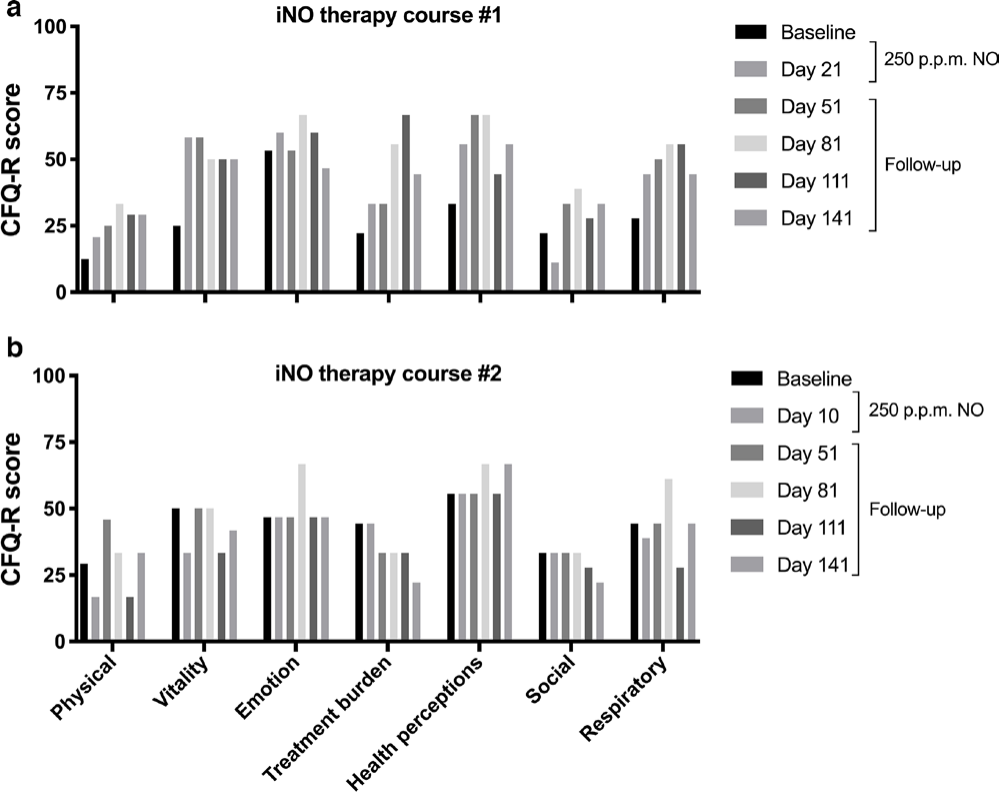
Cystic Fibrosis Questionnaire – Revised (CFQ-R). Selected domain scores from course #1 (a) and course #2 (b) of high-dose intermittent NO treatment are shown. Domain scores range from 0 to 100, with higher scores corresponding to higher quality of life. A minimal clinically important difference of 4.0 points has been determined in stable patients for the respiratory domain [26].

**Fig. 2.**
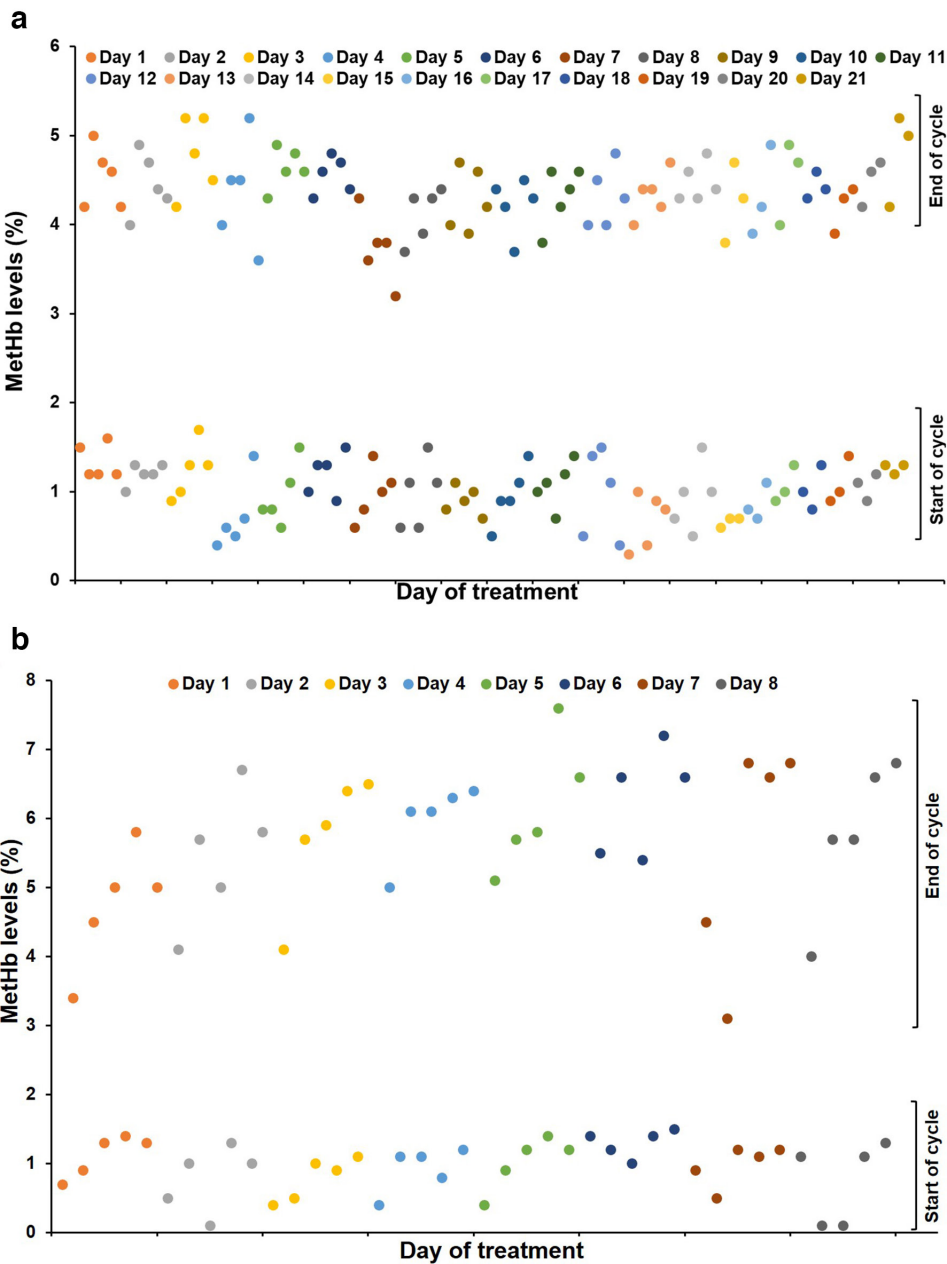
Methemoglobin levels. MetHb levels by treatment day from treatment course #1 (a) and #2 (b) measured at the start and end of each NO inhalation cycle (depicted by dots) using a pulse oximeter. MetHb levels increased after each inhaled NO cycle but returned to baseline levels following the period between intermittent NO inhalations. Levels did not exceed safety limits of <10%.

The patient completed the planned 3-week treatment course 1 with no significant adverse effects noted. In general, the patient noted improved respiratory symptoms and quality of life (Fig. 1a) and had small improvements in her lung function, 6MWD and inflammatory markers, but no significant change in heavily positive (4+) [32] acid-fast bacillus (AFB) smear and cultures for *M. abscessus* (Table 2). The patient had very reproducible and expected increases (range 0.3–5.2%) in MetHb (Fig. 2) during each dosing period that did not exceed the safety parameters of <10% [33]. During treatment course 1 for inhaled nitric oxide, the patient was treated with 160 p.p.m. NO five cycles/day (day 1–14) and for three cycles/day from day 15–21 (Fig. S2). As demonstrated in Fig. 2a, the levels of methemoglobin at the start of each treatment day were low (0.5–1.5%), which increased after each NO cycle (4–6%) but returned to normal levels following each cycle. No NO treatments were held or delayed due to elevated MetHb levels or inhaled and ambient NO_2_ levels (<2 p.p.m., not shown). Over the course of the follow-up period, the patient was able to lead a more normal life. Given the margin of safety with regard to MetHb and NO_2_ responses and overall tolerability, but no evidence of a microbiological response, the patient requested to repeat the treatment. A retreatment protocol was designed with an initial dose titration up to 240 p.p.m. and subsequent similar dosing schedule to the initial treatment.

**Table 2.** Selected efficacy measures – course #1 NO therapy

Course #1	Days	FEV1 (%)	FVC (%)	DLCO_**adj**_ (%)	6MWD (m)	6MWT (preSpO_**2**_%)	6MWT (postSpO_**2**_%)	CRP (mg dl^−1^)	ESR (mm h^−1^)	AFB stain [30]	AFB culture [30]	Time to (+) culture
Screen	Baseline	39	50	37	410	98	90	51	29	4+	4+	2 days/7 h
iNO	7	37	53		399	98	89	32	25	4+	4+	2 days/18 h
	14	40	59		379	98	91	14	18	4+	4+	2 days/4 h
	21	42	58	34	426	99	93	37	22	4+	4+	2 days/7 h
Follow-up	51	41	59		503	99	91	20	17	4+	4+	2 days/2 h
	81	42	60		459	100	95	35	34	4+	4+	2 days/9 h
	111	41	56		471	99	97	17	26	4+	4+	2 days/0 h
	141	38	58	40	362	99	94	18	28	4+	4+	2 days/2 h

FEV1, forced expiratory volume; FVC, forced vital capacity; DLCO_adj_, diffusion capacity of the lungs for carbon monoxide; 6MWD, 6 min walk distance; 6MWT, 6 min walk test; SpO2, peripheral capillary oxygen saturation; CRP, C-reactive protein; ESR, erythrocyte sedimentation rate; AFB, acid-fast bacillus.

#### Treatment course 2

The patient completed the initial two titration days through a dose of 240 p.p.m. without significant adverse effects. On day 3 the patient commenced NO inhalations with 240 p.p.m. for 30 min at 3.5 intervals five times daily. On days 3–6 the patient noted headaches and anxiety and on day 7 during NO inhalation, developed chest tightness, shortness of breath and anxiety associated with a decrease in oxyhaemoglobin saturation from 92–87%. The initial assessment for the day 7 events was that this was due to acute bronchospasm and nebulized albuterol was administered with resolution of symptoms and return to her baseline oxygenation status. On day 8, the patient noted anxiety and emotional lability and was unable to complete her scheduled 6MWT due to shortness of breath and desaturations down to 79% within 2–3 min of commencing the test. However, earlier on day 8, the patient was able to exercise with supplemental oxygen at her normal levels in pulmonary rehabilitation as per her daily routine. No further NO inhalations were administered and a workup for possible worsened pulmonary infiltrates, intercurrent pulmonary infection, pulmonary embolism, pulmonary hypertension or ventricular dysfunction was negative.

Review of MetHb levels showed that all preinhalation levels were <2% and peak MetHb remained within the protocol-specified 7–10% range (Fig. 2). However, peak MetHb levels ran about 2% higher than with previous 160 p.p.m. NO concentration. Inhaled NO_2_ levels remained <3 p.p.m. (max 2.9 p.p.m.; mean 2.6 p.p.m.). Quality of life assessment with the CFQ-R was done prior to discharge (day 10) and at her post-treatment follow-up visits and compared to baseline (Fig. 1b). Decreases in physical and vitality domains at day 10 recovered to baseline or higher by day 51. Selected efficacy measures were assessed at approximately 1 week into treatment and at the four monthly follow-up visits and compared to baseline (Table 3). 6MWD recovered to greater than baseline by day 51 and continued to increase at subsequent visits. Inflammatory markers mostly remained unchanged and there was no appreciable change in mycobacterial stain or culture quantity or in time to culture positivity. The patient completed the four planned monthly follow-up visits and the symptoms noted above gradually resolved or returned to baseline with no persistent sequelae noted.

**Table 3.** Selected efficacy measures – course #2 NO therapy

Course #1	Days	FEV1 (%)	FVC (%)	DLCO_**adj**_ (%)	6MWD (m)	6MWT (preSpO_**2**_%)	6MWT (postSpO_**2**_%)	CRP (mg dl^−1^)	ESR (mm h^−1^)	AFB stain [30]	AFB culture [30]	Time to (+) culture
Screen	Baseline	38	58	40	362	99	94	18	28	4+	4+	2 days/2 h
iNO	7	39	54		UTC	UTC	UTC	17	33	4+	4+	2 days/4 h
Follow-up	51	40	57		427	96	90	30	38	4+	4+	2 days/6 h
	81	42	57		479	98	92	25	29	4+	4+	2 days/9 h
	111	39	53		466	96	90	21	26	4+	4+	2 days/9 h
	141	40	57		471	95	90	5	26	4+	4+	2 days/3 h

FEV1, forced expiratory volume; FVC, vorced vital capacity; DLCO_adj_, diffusion capacity of the lungs for carbon monoxide; 6MWD, 6 min walk distance; 6MWT, 6 min walk test; SpO2, peripheral capillary oxygen saturation; CRP, C-reactive protein; ESR, erythrocyte sedimentation rate; AFB, acid-fast bacillus.

### *In vitro* susceptibility of *M. abscessus* to NO

**Fig. 3.**
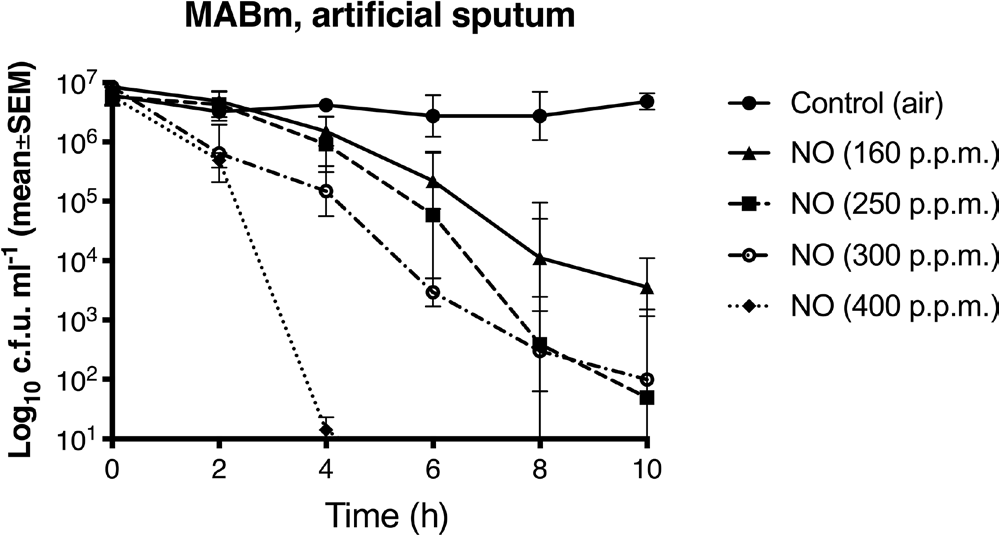
Dose–response effect of NO against *M. abscessus* subspecies *massiliense* in artificial sputum. A time–kill assay was performed using MAB_062600_1635 (early clinical infection strain, smooth colony) cultured in artificial sputum and treated with different concentrations of NO (0, 160, 250, 300, 400 p.p.m.). The means and standard error of mean of at least three independent experiments are shown. Significant differences from control were observed at 4 h for 160 and 250 p.p.m. (*P*≤0.01), at 2 h for 300 p.p.m. (*P*≤0.01) and at 2 h for 400 p.p.m. (*P*≤0.001).

In a dose–response study, continuous exposure to NO exhibited potent antimycobacterial activity against the early clinical course, smooth colony isolate MABm (MAB_062600_1635) at concentrations ≥160 p.p.m. MABm cultured in the control chamber (treated with humidified air only) did not show any loss of viability (Fig. 3). The time–kill curves showed over 3-log reduction after 10 h treatment with 160 p.p.m. NO and 100% kill after 4 h continuous exposure at the highest NO dose (400 p.p.m.). All subsequent experiments were performed at 250 p.p.m. NO as higher doses are not clinically relevant due to potential safety concerns in patients [24]. Similar NO anti-mycobacterial activity was observed in *M. abscessus* cultured on solid agar (Fig. S5).

**Fig. 4.**
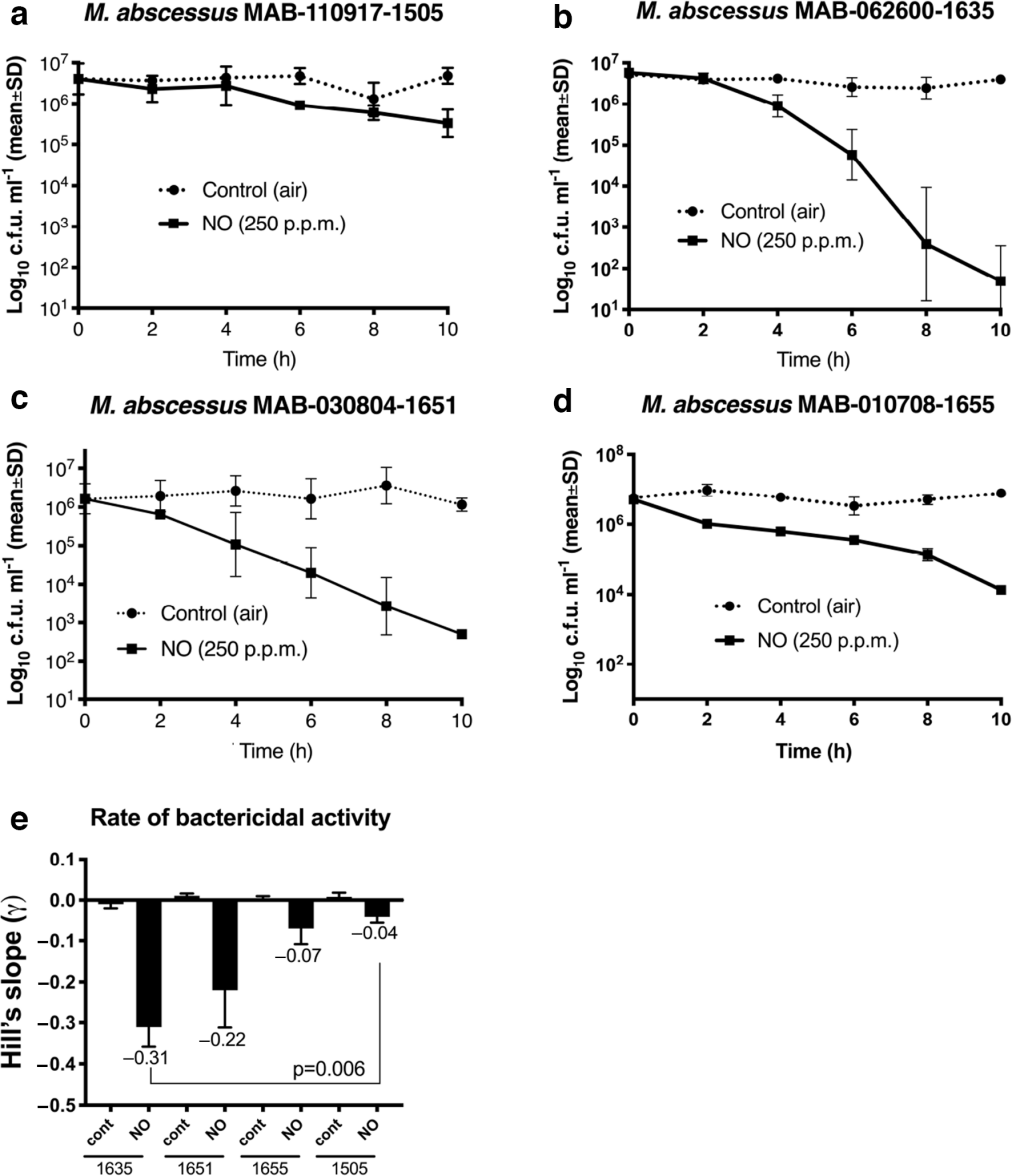
Time–kill curves of *M. abscessus* clinical isolates treated with NO. Survival curves for the patient’s strain (a) and comparison serial clinical isolates (b–d) are shown (at least three replicates). Bacteria cultured in artificial sputum were treated with humidified air (control) or 250 p.p.m. NO continuously for up to 10 h. (e) Mean Hill’s slopes for NO-treated clinical isolates were calculated using a nonlinear regression sigmoidal dose–response model. The Mann–Whitney test was used to calculate the *P* value between the patient’s strain (1505) Hill’s slope and that for the most susceptible *M. abscessus* comparison strain (1635).

As the accumulation of NO by-products can slowly acidify the culture media (Fig. S6a), we tested the effect of reduced pH in artificial sputum on *M. abscessus* viability. Acidification of artificial sputum as low as pH 5 did not affect the viability of *M. abscessus* strains in comparison to media at pH 7 (Fig. S6b–d). Time–kill assay against the patient’s isolate (MAB_110917_1505) revealed ≤1 log reduction after 10 h of exposure to 250 p.p.m. NO (Fig. 4a). The comparison serial clinical isolates (Table 1) demonstrated higher susceptibility to 250 p.p.m. NO with a 2–4 log reduction in 10 h (Fig. 4b–d). The Hill’s slope, representing the rate of killing, was significantly lower (*P*=0.006) in the patient’s isolate compared to the most susceptible comparison MABm isolate (Fig. 4e).

## Discussion

The clinical treatment protocol demonstrated that intermittent high-dose NO treatment at 160 p.p.m. was tolerated by the patient but did not achieve eradication of *M. abscessus* lung infection after 21 days. This lack of antibacterial effect at 160 p.p.m. coupled with the improvements noted in quality of life, lung function and functional status, as reflected by increased 6MWD, contributed to a desire to repeat the treatment at a higher concentration of inhaled NO. The reasons for the patient’s symptoms and eventual need to stop treatments at 240 p.p.m. remain unclear. Increased anxiety, headache, dyspnea on exertion and gas exchange abnormalities have been reported to occur due to buildup of methemoglobin (in the range of 20–30%) or nitrogen dioxide from continuous high concentration NO delivery [34–36]. These effects were not seen with the administration of NO at 160 p.p.m. for this patient or others treated under similar protocols [22–24]. The severity of symptoms was greater than expected, given that the patient was within the designated exposure limits for methemoglobin and nitrogen dioxide exposure; however, the patient had a chronic anaemia, which has been reported to accentuate the adverse effects of methemoglobin at lower levels [33, 37]. A workup to identify alternative aetiologies included CT chest, sputum microbiology, inflammatory markers (CRP, ESR, white blood cell count), a d-dimer (a fibrin degradation product indicative of thromboembolic disease when elevated) and pro-BNP (brain natriuretic protein, as a marker for increased atrial stretch indicative of change in right heart pressures) were all within normal limits or unchanged from baseline. The patient had a known history of headaches and anxiety, which may have worsened without direct relationship to NO inhalation. Wheezing was heard with the patient’s initial respiratory event during NO inhalation. The patient was administered albuterol, leading to decrease in wheezing and increase in oxygen saturation. The possibility of bronchospasm related at least in part to the patient’s significant underlying airway disease remains.

The *in vitro* NO susceptibility studies provide evidence for dose-dependent anti-mycobacterial activity of high-dose NO and heterogeneity in susceptibility to NO treatment between different *M. abscessus* clinical strains. Clonal diversification in the serial comparison strains over time in the human host, which may lead to changes in characteristics such as colony morphology, nutrient utilization and antibiotic resistance, may also lead to factors that increase resistance to NO [28, 38]. The fact that this patient’s *M. abscessus* strain was the least susceptible to NO relative to the comparison MABm strains is consistent with her lack of microbiological response to short-term inhaled NO. Longer treatment regimens that may include home administration could be required to see reduction or eradication of *M. abscessus* lung infection.

A growing number of studies have explored the potential application of exogenous NO therapy to enhance mycobacterial killing, primarily in *Mycobacterium tuberculosis* (Mtb) preclinical models. Compounds that stimulate intracellular production of NO, such as pretomanids (e.g. bicyclic nitroimidazoles) have shown Mtb-killing activity [39, 40]. Furthermore, NO donor compounds such as DETA/NO, S-nitrosothiols, and furoxan derivatives have also demonstrated anti-Mtb activity in preclinical models [41–43]. Despite the efficacy in preclinical models, there has been limited evidence on the efficacy of NO donor compounds in the clinical setting. Some of the key challenges that hinder clinical application of current NO donors include capacity for steady and prolonged release of high-dose NO, reaching the target pathogen within airway mucus and sputum, and the requirement for a low-pH environment (acidic conditions) for optimal release of NO. In this study, we performed an *in vitro* time kinetic experiment to measure the antibacterial activity of NO against clinical strains of *M. abscessus* exposed to gaseous nitric oxide for different time points up to 10 h. A potential limitation of translating this therapy to patients is the long duration of gaseous NO at high doses and associated side effects. In a previous 48 h *in vitro* experiment (data not shown), we observed that continuous exposure and intermittent exposure for the same total time and concentration of NO exposure results in similar activity against *M. abscessus *. During patient treatment, we were able to provide 2.5 h of NO in a day (30 min cycle, five times a day) at 160 p.p.m., and a total of 17.5 h of NO at 160 p.p.m. in a week. The hope is that the cumulative concentration–time dose will be sufficient to kill the mycobacteria without the toxicity seen with prolonged continuous exposure to these very high concentrations of NO; however, it is not certain that the relationship between exposure time, concentration of NO and total dose in our *in vitro* experiments and clinical delivery will have the same efficacy. The long-term effects of NO may include oxidative effects in the lung, decreased neutrophil accumulation and surfactant function, and peroxynitrite generation leading to genotoxic alterations [44], which could potentially limit this approach.

Inhaled NO gas is an FDA-approved treatment in infants at concentrations below 80 p.p.m. with an outstanding safety and tolerability record [45]. To bypass the challenges of developing inhaled NO-releasing compounds for clinical use, our group and others have investigated the antibacterial properties of NO gas. High-dose NO (160 p.p.m.) shows potent antimicrobial activity against a variety of bacteria, viruses and fungi *in vitro* and *in vivo* in a rat model of *Pseudomonas aeruginosa* lung infection [18–20, 46]. NO and other reactive nitrogen intermediates (RNI) can exert anti-mycobacterial activity by multiple mechanisms, including alterations in DNA and interaction with Fe/S clusters of bacterial enzymes, resulting in enzymatic inactivation and oxidative damage to membrane lipids [8]. However, mycobacteria have evolved to develop innate protection against NO anti-mycobacterial activity. Studies using a *Mycobacterium smegmatis* mutant that is unable to produce mycothiol, the thiol analogue of glutathione, have shown that high levels of mycothiol provide protection against damage from NO and RNI in mycobacteria [47]. The authors suggest that higher doses and longer duration of NO exposure could overcome this protective mechanism in mycobacteria. The *M. abscessus* strains tested in our study exhibited higher resistance to high-dose NO (250 p.p.m.) than previously reported strains of *M. smegmatis *, which showed 100% kill after 10 h exposure to 160 p.p.m. NO [47]. The link between mycothiol levels and *M. abscessus* susceptibility to NO and a potential therapeutic role for blocking this pathway as a means of enhancing the antimycobacterial effect of NO requires further investigation.

Human trials on the benefits of inhaled NO therapy in patients with pulmonary NTM infection are limited. Long *et al*. reported safe administration of 80 p.p.m. inhaled NO for a period of 72 h in patients with pulmonary Mtb, but failed to demonstrate improvement in bacterial clearance at this dose [48]. This prompted other investigators to examine the safety of high-dose NO in adults. A pilot clinical study of intermittent (30 min cycles, five times daily, 3 weeks) inhaled 160 p.p.m. NO in healthy adults found no adverse events and concluded that administration of high-dose NO was safe and well tolerated [21]. A subsequent phase I clinical study in CF patients with chronic bacterial lung infection confirmed the safety of intermittent high-dose inhaled NO therapy at 160 p.p.m. and revealed significant mean reductions in airway sputum bacterial load, including reductions in *M. abscessus,* in two patients. However, the study failed to show complete eradication of *M. abscessus* in these patients [22]. A recent pilot study of nine chronic pulmonary *M. abscessus* patients treated with intermittent 160 p.p.m. NO did not achieve culture conversion following 21 days of treatment, but demonstrated reductions in sputum bacterial load in a subset of patients [24]. The differential susceptibility of *M. abscessus* clinical isolates to 250 p.p.m. NO reported here suggests that higher concentrations and longer duration of inhaled NO could be required to achieve complete eradication in patients with highly resistant strains. Furthermore, a number of studies show that NO can potentiate antibiotics (e.g tobramycin, gentamicin) against bacterial biofilm [49] or Gram-negative and -positive bacteria [50]. Further *in vitro* studies are required to assess potential synergy between NO and anti-NTM antibiotics in order to maximize the bactericidal activity of NO in chronic NTM disease.

In conclusion, our findings presented here demonstrate the anti-mycobacterial activity of high-dose NO against several clinical isolates of *M. abscessus in vitro* and support further evaluation of inhaled NO as a novel therapy in patients with refractory NTM lung infection.

## Supplementary Data

Supplementary material 1
