# Supplementary material for: Antibacterial activity of high-dose nitric oxide against pulmonary Mycobacterium abscessus disease
Source: Access Microbiol. 2020 Aug 10;2(9):acmi000154. doi: 10.1099/acmi.0.000154 (PMC7656188; doi:10.1099/acmi.0.000154)
Supplement: Supplementary material 1 [file acmi-2-154-s001.pdf]

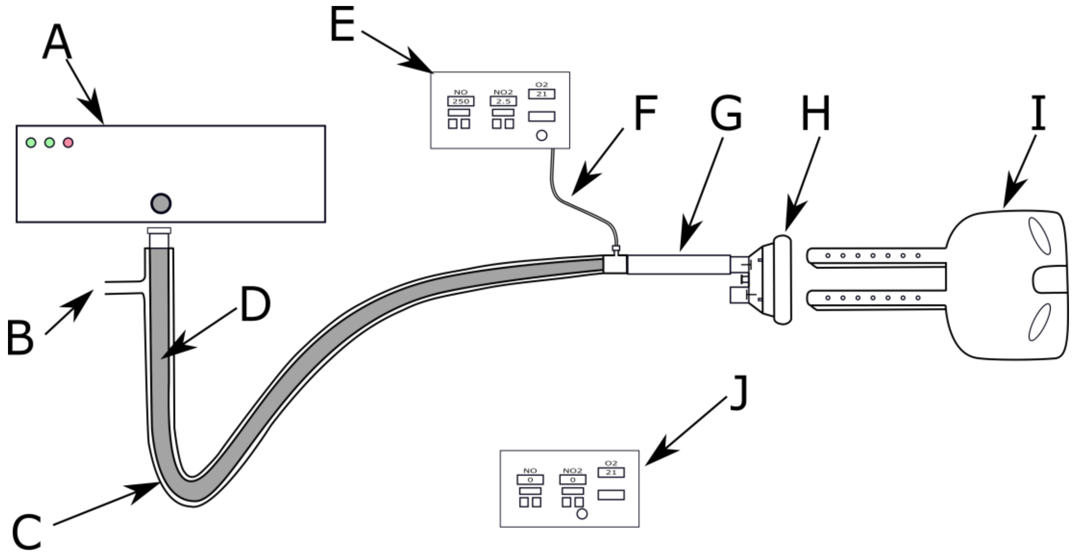

Fig. S1. Nitric Oxide Generator and Delivery Circuit. A: NO generator; B: exhaust port; C: outer (reservoir) limb; D: inner (delivery) limb; E: delivery gas monitor; F: sample line; G: 6” extension tube; H: bi-valve mask; I: silicone head gear; J: environmental monitor.

A. Inhaled NO Course 1

Days 1-14: 160 ppm inhaled NO for 30 minutes every 3-4 hours daily (5 times/day)

↓

Days 15-21: 160 ppm inhaled NO for 30 minutes every 6 hours daily (3 times/day)

B. Inhaled NO Course 2

Day 1: 160 ppm inhaled NO for 30 minutes, followed by 10 ppm increase up to 200 ppm, for subsequent doses administered every 3-4 hours (total 5 times/day)

↓

Day 2: 200 ppm inhaled NO for 30 minutes, followed by 10 ppm increase up to 240 ppm, for subsequent doses administered every 3-4 hours (total 5 times/day)

↓

Days 3-21: 240 ppm inhaled NO for 30 minutes delivered every 3-4 hours (5 times/day)

Fig. S2. Flow Diagram Demonstrating Nitric Oxide Delivery During the Treatment. A: Schedule for first inhaled nitric oxide course for 3 weeks. B: Schedule for second inhaled nitric oxide course for 3 weeks with increasing doses for Day 1 and 2.

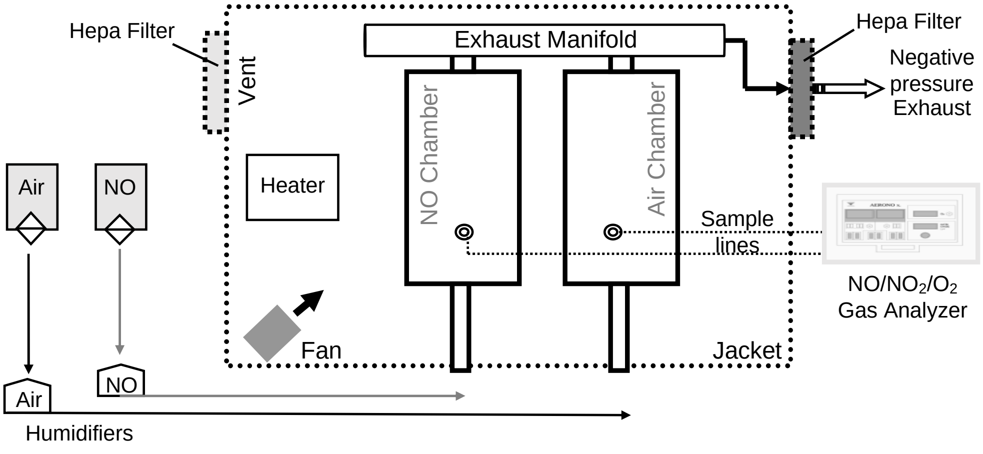

Fig. S3. Schematics of the in vitro NO exposure chamber. NO gas from cylinder (or NO Generator) is delivered at precise concentration to a humidifier before entering the NO Exposure Chamber. Compressed air pump is used to deliver humidified air to the chamber for the control group. An AeroNOx Gas Analyzer constantly samples air from NO Chamber and room environment.

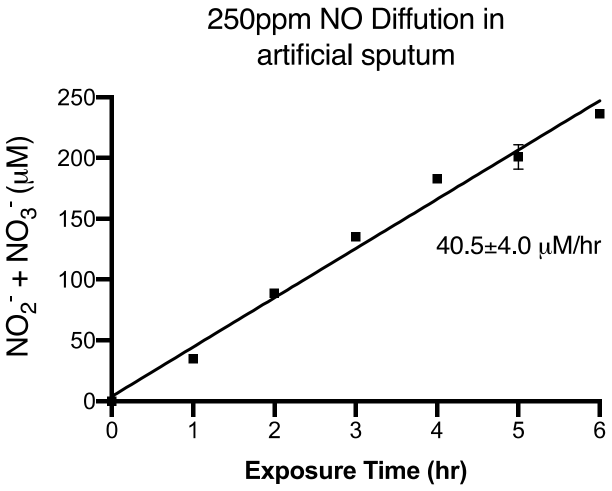

Fig. S4. NO delivery rate in artificial sputum. The uptake of NO in artificial sputum was measured by continuously treating 2 mL of media with 250ppm NO inside the NO Chamber. NO uptake was analyzed over time for NO_2/NO_3 levels using Griess reagent assay. The graph shows a constant and linear uptake of NO inside the exposure chamber.

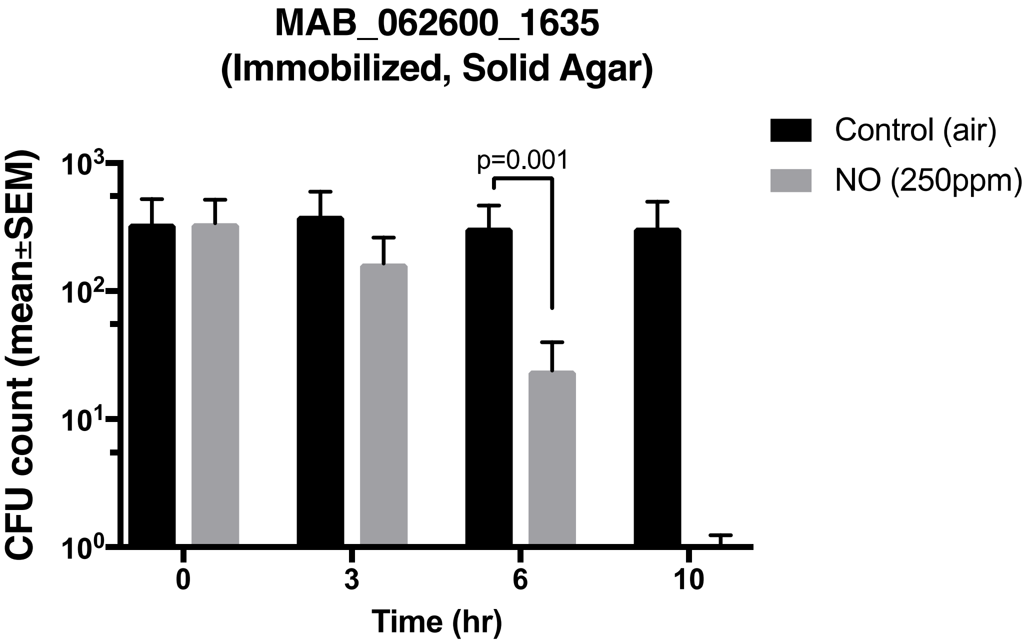

Fig. S5. Antibacterial activity of high-dose NO against *M. abscessus* cultured on solid agar. *M. abscessus* susceptibility to 250ppm NO was tested after spreading ~10^3 CFU bacteria on Middlebrook 7H11 agar and exposing to continuous NO treatment inside the NO Chamber. p value was calculated by unpaired t-test (at least 3 replicates).

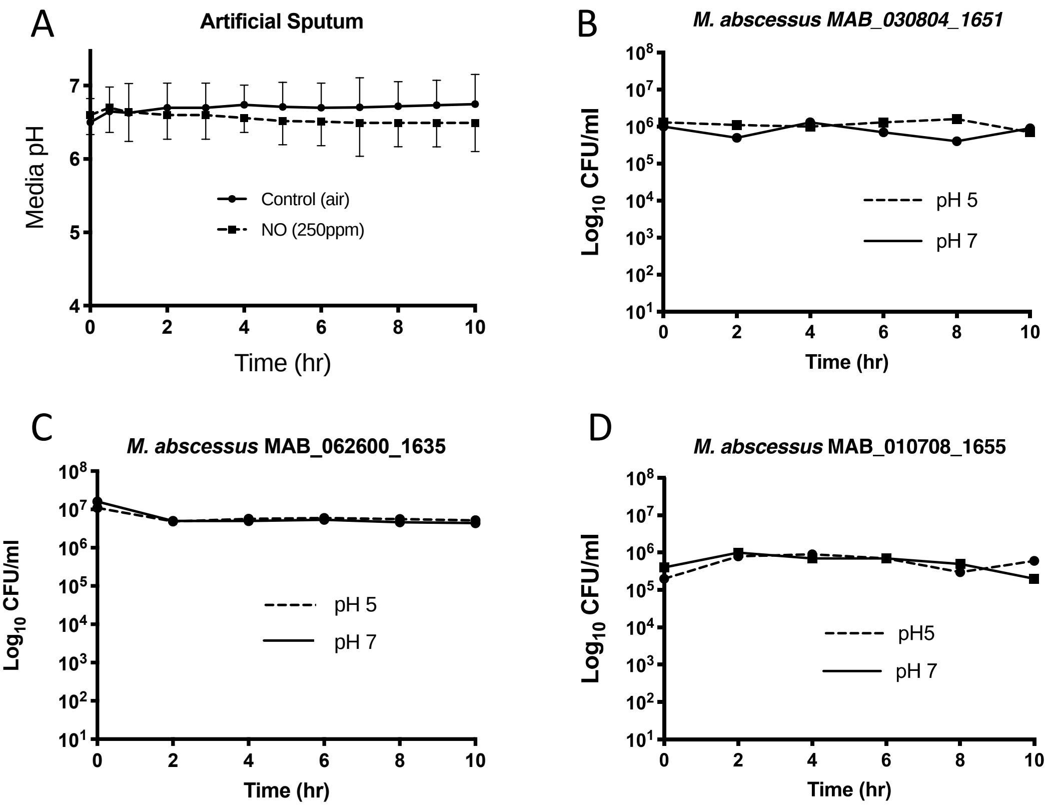

Fig. S6. Effect of pH on *M. abscessus* viability in artificial sputum. **Panel A**: treatment with 250ppm NO continuously for up to 10hr results in minute decrease in culture media pH. **Panels B-D**: to account for the effect of reduced pH on *M. abscessus* viability inside NO Chamber, various strains were cultured in artificial sputum at reduced pH 5 and compared to pH 7. No significant difference was observed between bacterial viability in artificial sputum pH 5 and pH 7.
